# A pilot randomized controlled trial of the effects of dietary nitrate supplementation on dynamic cerebral autoregulation in older adults with metabolic syndrome

**DOI:** 10.14814/phy2.71073

**Published:** 2026-08-25

**Authors:** Jigar Gosalia, Jocelyn M. Spicuzza, Christine Bowlus, Annabelle Brinkerhoff, Stephen R. Baker, Juan Jan Qiu, Daniel B. Kim‐Shapiro, Jim A. Pawelczyk, David N. Proctor

**Affiliations:** ^1^ Department of Medicine, Division Renal Diseases and Hypertension The University of Colorado Anschutz Medical Campus Aurora Colorado USA; ^2^ Department of Psychiatry The University of Pittsburgh School of Medicine Pittsburgh Pennsylvania USA; ^3^ Department of Kinesiology The Pennsylvania State University University Park Pennsylvania USA; ^4^ College of Medicine The Pennsylvania State University University Park Pennsylvania USA; ^5^ Department of Physics and Translational Science Center Wake Forest University Winston‐Salem North Carolina USA

**Keywords:** cerebral autoregulation, cognition, dietary nitrate, functional near infrared spectroscopy, metabolic syndrome

## Abstract

Metabolic syndrome (MetS) is associated with reduced nitric oxide (NO) bioavailability and impaired cerebrovascular function, potentially increasing cognitive decline risk. Dynamic cerebral autoregulation (dCA) maintains cerebral blood flow and may involve NO‐related vascular mechanisms. Whether dietary nitrate supplementation influences dCA in MetS remains unclear. This pilot trial evaluated feasibility and explored effects of nitrate supplementation on dCA. Twenty‐two older adults with MetS (55–75 years) were randomized in a double‐blind, placebo‐controlled design to receive nitrate‐rich beetroot juice (BRJ; 6.4 mmol NO_3_^−^·day^−1^) or nitrate‐depleted placebo for 4 week. dCA was assessed using transfer function analysis (TFA) of beat‐to‐beat blood pressure and functional near‐infrared spectroscopy‐derived cerebral oxygenation. Primary analyses examined frontal dCA holistically, with exploratory regional and cognitive outcomes. dCA was indexed by TFA phase and gain. The intervention was well tolerated, adherence was high, and BRJ increased plasma nitrate concentrations versus placebo (*p* < 0.001). No significant effects were observed for frontal dCA or cognition. Exploratory analyses indicated region‐specific differences in dCA in select frontal areas, characterized by improvements in TFA phase and gain. This pilot trial demonstrates the feasibility of dietary nitrate supplementation in MetS. While no effects were observed in primary outcomes, exploratory analyses suggest potential region‐specific improvements in dCA.

## Graphical abstract

4‐weeks of nitrate‐rich beetroot juice is feasible, tolerable, and may confer region‐specific improvements in dynamic cererbral autoregulation in adults with metabolic syndrome.

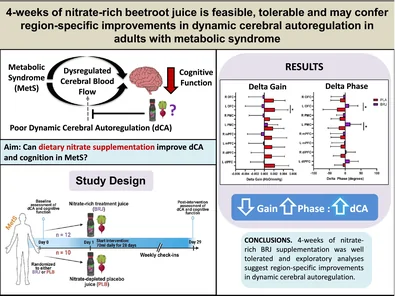

## 1 INTRODUCTION

Individuals with metabolic syndrome (MetS) demonstrate worse cognitive functioning, specifically in cognitive domains regulated by frontal regions such as executive functioning (Marseglia et al., 2021; Segura et al., 2009), and dementia (Beeri & Gu, 2024; Qureshi et al., 2024). Currently, there are no cures for dementia, highlighting the importance of early preventive and mechanistic intervention studies in MetS progression. However, the optimal timing and efficacy of interventions targeting cerebrovascular function in this population remain unclear.

In older adults with MetS, cerebral microvascular dysfunction may be driving structural deterioration of pivotal brain regions responsible for carrying out executive functions (van Sloten et al., 2020; Zlokovic et al., 2020). Dynamic cerebral autoregulation (dCA) is an important mechanism of cerebrovascular blood flow regulation and serves as a protective mechanism for the brain against large fluctuations in blood pressure that can result in hyper‐ and hypo‐perfusion of vulnerable brain regions (Claassen et al., 2021). Individuals with poor glycemic control and obesity, which are key characteristics of MetS, demonstrate disrupted dCA (Coucha et al., 2018). Importantly, impaired dCA has been reported in patients with mild cognitive impairment and dementia (Marshall, 2012). Impaired dCA may be a primary mechanism contributing to increased risk of cognitive decline in MetS and can be targeted therapeutically. Blood pressure maintenance through dCA is achieved by alterations in peripheral vascular resistance primarily through changes in microvascular tone, and cerebral microvascular dysfunction is a significant contributor to impairments in dCA (Claassen et al., 2021). Therefore, the cerebral microvasculature represents a relevant system that can be targeted in MetS to improve dCA and cognition.

Nitric oxide (NO) bioavailability is a significant contributor to dCA and plays an important role in the cerebral circulation, both as a signaling molecule and potent vasodilator (White et al., 2000). Reduced NO bioavailability promotes inflammation, oxidative stress, and endothelial dysfunction, all of which negatively impact cerebral microvascular function and subsequent dCA (Deanfield et al., 2007; Delgado Spicuzza et al., 2024; Mcnally et al., 2016; Picón‐pagès et al., 2019; Seals et al., 2011; Stephan et al., 2017). Recent evidence shows that consumption of nitrate‐rich foods, such as beetroot juice (BRJ), augments systemic levels of NO via the enterosalivary nitrate‐nitrite‐nitric oxide (NO3− → NO2− → NO) pathway (Clifford et al., 2015).

Previous investigations suggest that an acute dose of BRJ (5.0–24.2 mmoL) increases resting cerebral blood flow, as measured by transcranial doppler ultrasound (Chirinos et al., 2017; Curry et al., 2016) and near infrared spectroscopy (Thompson et al., 2015; Wightman et al., 2015), in both healthy young adults and older heart failure patients. However, the effects on dCA remain uncertain, particularly in older adults with cardiometabolic risk. Few studies have assessed whether nitrate‐rich BRJ can improve dCA; Two studies have assessed the effects of BRJ on dCA in healthy young adults, one study demonstrated improvements in dCA after 7 days of BRJ supplementation (Fan et al., 2019) whereas the other found no significant effects of BRJ on dCA (Horiuchi et al., 2022). Horiuchi and colleagues indicated that their lack of significant findings may be a result of studying a healthy sample and suggested that nitrate‐rich BRJ supplementation may be more effective in improving dCA in older adults with CVD risk factors (e.g., MetS) (Horiuchi et al., 2022). Additionally, previous investigations suggest that 4 weeks of beetroot juice supplementation may be an effective timeframe to observe improvements in vascular (Jones et al., 2019) and cognitive function (Gilchrist et al., 2014). Thus, 4 weeks of nitrate‐rich BRJ supplementation represents a potential dietary approach to improve dCA in MetS; however, it has yet to be established. Previous investigations of dCA have often utilized transcranial doppler or MRI‐based measurements to quantify cerebral blood flow. However, these measurements largely represent macrovascular function, and do not provide much information about cerebral microvasculature function. Functional near‐infrared spectroscopy (fNIRS)‐derived oxyhemoglobin [HbO] signal can be used as an index of microvascular blood flow to quantify dCA that reflects the microvasculature (Herold et al., 2018; Scholkmann et al., 2014).

The primary aim of this pilot double‐blind, placebo‐controlled, randomized parallel arm clinical trial was to evaluate the feasibility of 4 weeks of nitrate‐rich beetroot juice supplementation (BRJ) in older adults with MetS and to explore its associations with dCA compared to nitrate‐depleted placebo (PLB). The secondary aim was to explore differences in executive cognitive performance between BRJ and PLB, and to examine associations between changes in dCA and executive function. An exploratory aim was to analyze the effects of supplementation on dCA for various frontal regions individually to determine if improvements were regionally specific. Given the modest sample size, all outcome analyses were considered exploratory and hypothesis‐generating.

## 2 METHODS

### 2.1 Participants

Study participants were recruited from the Pennsylvania State University campus and the greater surrounding State College, PA community, and provided written informed consent to participate in this registered clinical trial (NCT05532423). A total of 40 participants were phone‐screened, of which 28 participants were further screened in person. A total of 22 participants were eligible and completed the study. This study was approved by the institutional review board at The Pennsylvania State University (IRB #00020830). All procedures were approved by the Office of Research Protections at The Pennsylvania State University in agreement with the guidelines set forth by the Declaration of Helsinki.

**FIGURE 1:**
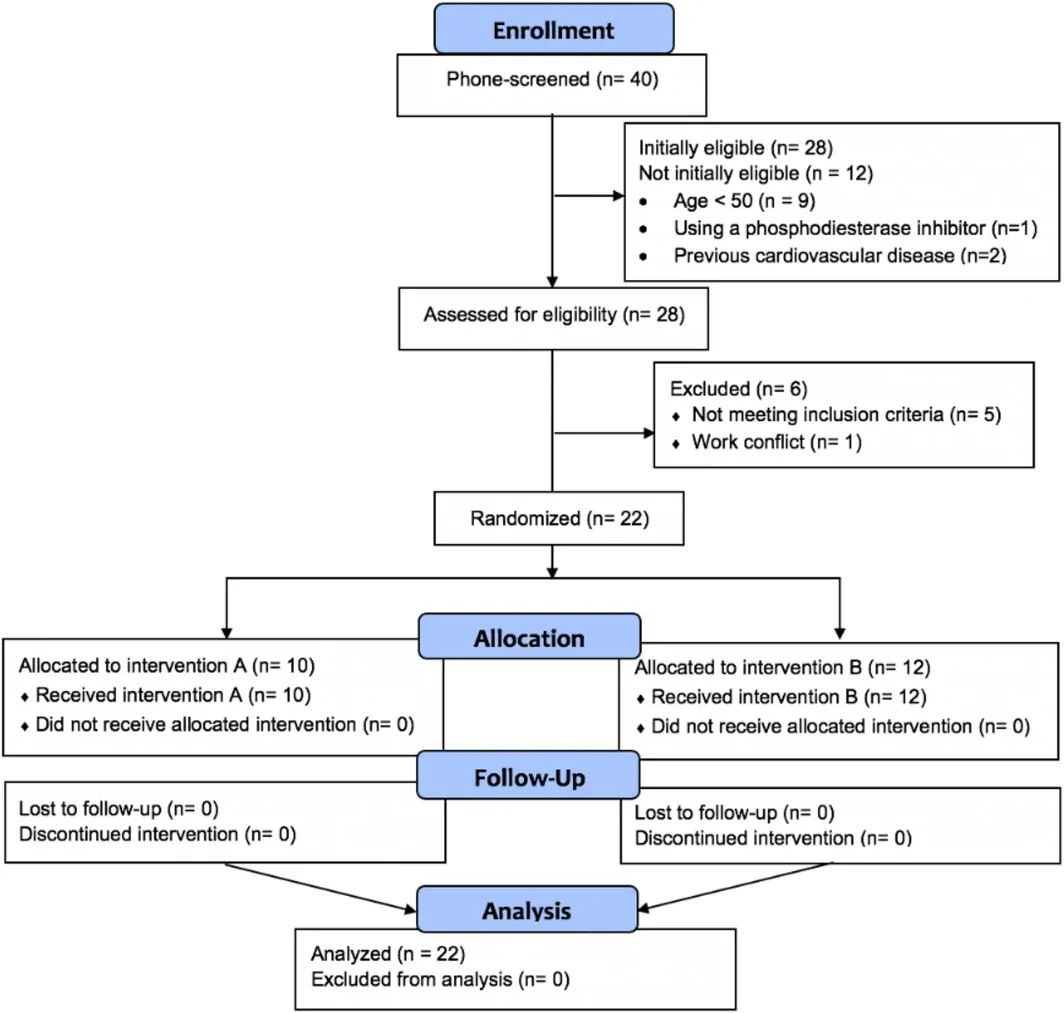
Study CONSORT flow diagram. A summary of the chronic randomized controlled parallel arm trial to assess the influence of nitrate‐rich and depleted beetroot juice supplementation on cerebrovascular and neurocognitive function. (a) Nitrate‐depleted beetroot juice (PLA) and (b) Nitrate‐rich beetroot juice (BRJ).

Community dwelling older men and women aged 55–75 years old were screened for MetS for enrollment in this clinical trial. Inclusion criteria were selected to identify individuals with metabolic syndrome at elevated cardiometabolic risk. MetS status was determined using The National Cholesterol Education Program (NCEP) Adult Treatment Panel III (ATP III) definition, indicating the presence of three or more of the five cardiometabolic disease risk factors: [(1) systolic blood pressure >130 mmHg OR diastolic blood pressure >85 mmHg; (2) Fasting plasma glucose >100 mg/dL; (3) Triglyceride count >150 mg/dL; (4) HDL‐c (men <40 mg/dL; women <50 mg/dL); (5) Waist circumference (men >102 cm; women >88 cm)]. Individuals with ≥3 components were classified as MetS and eligible for the study. Additional screening items included the mini mental state examination (MMSE) and the beck depression inventory (BDI‐II) for clinically relevant cognitive impairment and depression. Participants were excluded from the study if they presented with overt cardiovascular, metabolic, hematologic, pulmonary, renal, musculoskeletal, or neurological disease, cancer, uncorrected visual impairments, tobacco use, and BDI‐II scores >14, or MMSE <24. For women, only postmenopausal women (last menses >1 year as indicated by self‐report) were recruited to reduce the confounding effects of hormonal status. Lastly, individuals on the following medications at the time of screening were excluded: hormone replacement, nitrates, phosphodiesterase inhibitors, and long‐term anti‐inflammatory drug usage. An overview of the study flow CONSORT diagram (Figure 1) is provided.

### 2.2 Sample size considerations

This study was designed as a pilot randomized controlled trial; therefore, a formal a priori power calculation was not performed. However, a previous pilot investigation that explored the effects of 4 weeks of beetroot juice supplementation on vascular function in a parallel‐arm design included a total sample size of *n* = 18 and saw significant improvements in brachial artery flow mediated dilation compared to placebo (Jones et al., 2019). The target sample size was based on feasibility, resource availability, and consistency with prior pilot studies assessing vascular outcomes and BRJ supplementation. The final sample size of the current study (*n* = 22) reflects participants who completed all study procedures and were deemed sufficient to evaluate feasibility, estimate variability, and generate preliminary effect size estimates to inform future adequately powered trials.

### 2.3 Study design

**FIGURE 2:**
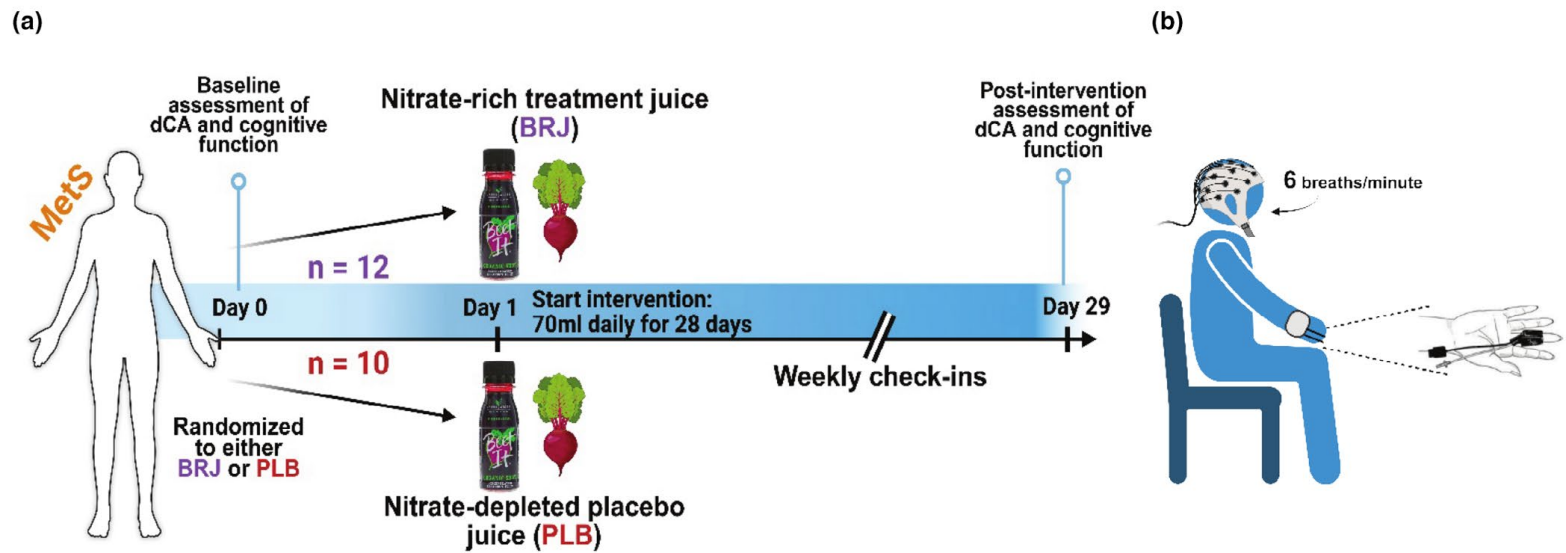
Experimental design for this double‐blind randomized placebo‐controlled clinical trial. (a) Study timeline and design; (b) Experimental setup for dCA assessment. An fNIRS cap with sources and electrodes was fitted on the participants head (NIRScout, NIRx) for measurements of oxygenated (HbO) and deoxygenated (HbR) hemoglobin. A finger blood pressure cuff (Finometer MIDI, Finapres Inc.) was wrapped around the middle finger on the left hand for beat‐to‐beat blood pressure measurements. Participants breathed at a rate of 6 breaths/min (5 s inhalation/5 s exhalation) for 6 min while HbO and arterial blood pressure were continuously recorded to assess dCA using transfer function analysis.

A schematic of the experimental protocol for this double‐blind, randomized, placebo‐controlled parallel arm clinical trial (Figure 2a) is provided. In brief, participants completed a baseline visit for assessment of cognitive function and dCA. Participants arrived between 7 and 10 am having met the following pre‐visit conditions for all study visits: 12 h fasting from food and caffeine, 48 h without alcohol and dietary supplements, and 24 h refraining from vigorous exercise. Participants were seated and instrumented for assessment of dCA, followed by an additional rest period of 10 min. Following dCA assessment, a baseline venous blood draw was then taken from the left arm for measurement of plasma nitrate and nitrite concentrations. Lastly, participants completed a neurocognitive battery. At the end of the baseline study, participants were randomized for daily consumption (70 mL) of either NO_3_^−^‐rich (BRJ, 300 mg NO_3_^−^ in 70 mL/6.4 mmol Beet‐It Organic, James White Juice Company) or NO_3_^−^‐depleted (PLB, 40 mg NO_3_^−^ per 70 mL/0.38 mmol nitrate‐depleted Beet‐It Organic, James White Juice Company) beetroot juice for 4 weeks, prior to a final experimental visit. Randomization was performed using a computer‐generated allocation sequence and participants were assigned in a 1‐to‐1 ratio to one of two randomization sequences by CRC nurse staff. Randomization was not stratified by sex or cardiometabolic variables. Blinding was maintained by using juices with identical taste, ensuring that both participants and investigators remained unaware of the treatment assignments.

During the 4‐week supplementation period, participants were asked to avoid antibacterial mouthwashes and toothpastes while participating in the study to minimize reductions in nitrate‐reducing commensal bacterium in the oral cavity. At 24 h following the final juice consumption, participants returned for an end of study visit, which was identical to the baseline visit. Compliance of juice consumption was verified by having participants return their empty bottles and verbal confirmation of consumption at the final visit and assessed as total number of bottles returned/total number of bottles given. In addition, the palatability and acceptability questionnaire was administered at the final visit. The blood sample in the follow‐up visit was taken approximately 24–28 h after the last BRJ dose to test the chronic effects of beet juice consumption.

### 2.4 Feasibility and acceptability outcomes

The study was designed to evaluate feasibility of the intervention protocol and data collection procedures. Feasibility was assessed by compliance with the intervention in the active study participants and total number of completed participants who were enrolled into the study. In addition, acceptability and palatability of the 4‐week beetroot juice intervention was assessed through a questionnaire administered at end of study. The palatability and acceptability questionnaire was administered through Penn State Qualtrics and involved several questions that asked participants to provide their subjective thoughts on consuming beetroot juice for 4 weeks. The questions asked about their likeness to the qualities of the drink (taste, smell, texture, aftertaste, etc.), taste profile of the drink (sweetness, sourness, and bitterness), likelihood of drinking BRJ after study completion, purchasing the juice, and adverse events or side effects related to juice consumption. These questions were asked through slider questions using point scales, yes/no questions, multiple choice responses, and open‐ended questions to gather both qualitative and quantitative data regarding the palatability and acceptability of translating beetroot juice clinically. The palatability scores were rated on a scale from 0 to 10, in which 0 was rated as highly unpleasant, 5 was rated as neutral, and 10 was rated as highly pleasant.

### 2.5 Transfer function analysis for assessment of dCA

dCA was measured using transfer function analysis (TFA) with arterial blood pressure (ABP) measured by finger photoplethysmography (Finometer MIDI, Finapres Inc.) as the input and cerebral blood flow (CBF) as indexed by HbO from functional near‐infrared spectroscopy (fNIRS) measurements (NIRScout, NIRx) as the output. The theory behind fNIRS for relative measurements of oxygenated (HbO) and deoxygenated (HbR) hemoglobin, as indices of cerebral blood flow, has been detailed previously (Gosalia et al., 2025; Pinti et al., 2020). The fNIRS‐derived HbO signal was chosen to be used as an index of microvascular cerebral blood flow over other methods such as transcranial doppler because the HbO signal better reflects the microvasculature (Pinti et al., 2020).

TFA involves linear, stationary modeling and a fast Fourier transform algorithm to compute spectral estimates of blood pressure and fNIRS‐derived cerebral blood flow. Intact autoregulation minimizes the influence of blood pressure on cerebral blood flow by attenuating the propagation of pressure waveforms at lower frequencies (<0.2 Hz) through actions on vascular tone. TFA allows for the derivation for gain and phase‐shift at each frequency, which are key parameters of dCA measured with TFA. Gain reflects the compression of brain blood flow velocity amplitude changes in response to blood pressure. Lower gain values indicate better compression of brain blood flow velocity amplitude changes in response to blood pressure. Phase‐shift represents the time lag between blood pressure and brain flow velocity at a given frequency, represented in degrees or radians. Larger phase‐shifts indicate that dCA properly buffers the cerebrovasculature from changes in blood pressure. In addition to gain and phase‐shift, coherence was measured to test the linearity between the waveforms of blood pressure and fNIRS‐derived cerebral blood flow. For TFA, a coherence >0.35 was considered acceptable to indicate linearity for assessing and interpreting TFA gain and phase (Panerai et al., 2023). Measurement of dCA through TFA involves perturbation of ABP to track corresponding changes in fNIRS‐derived HbO signal.

#### 2.5.1 Functional near infrared spectroscopy (fNIRS) and finometer setup

For fNIRS measurements, the continuous wave NIRScout system (NIRx Medical Technologies LLC, Glen Head, NY) was utilized to measure hemodynamic changes using an applied montage consisting of 14 double tip light source electrodes and 13 detector electrodes to record hemodynamic signals in the cerebral cortex. Source electrodes were emitted at 760 nm and 850 nm. Source‐detector pairs were chosen based on regions of interest utilizing the fOLD software (v2.2) that employs EEG anatomical landmarks from the international 10–20 system to correspond to underlying regions of interest (Zimeo Morais et al., 2018). For this study, interest was in frontal regions (i.e., dorsolateral prefrontal cortex [dlPFC], medial prefrontal cortex [mPFC], orbitofrontal areas [OFC], and the anterior portions of the premotor regions of the brain [PMC]) because MetS exhibits poorer frontal lobe regulated cognitive abilities (Falkowski et al., 2014). There was a total of 40 source‐detector pairings creating 40 separate channels with an average separation distance of 3.5 cm (range: 2.9–4.0 cm) for the ROI [dlPFC (8 channels; mean SDD: 35.3 mm), mPFC (12 channels; means SDD: 35.2 mm), OFC (8 channels; mean SDD: 34.0 mm), and the PMC (14 channels; mean SDD: 36.8 mm)]. For the primary analysis, all 40 channels encompassing these frontal regions were averaged to encompass a global frontal lobe value. For exploratory analyses, channels were averaged to correspond to the specific underlying cortical region as mentioned above. Details about the fNIRS calibration, signal quality determination, and setup can be found in File S1.

Measurements of arterial blood pressure (ABP) were taken using finger photoplethysmography through the finometer MIDI which utilizes an inflatable finger cuff, a photoplethysmography sensor, and a pressure controller unit (Fantini et al., 2016). The finger cuff was fitted on the middle finger for each participant and signals were auto calibrated for a minimum of 5 min to ensure a stable reading. ABP was recorded using Labchart sampled at a frequency of 1000 Hz. The testing room was kept at a constant temperature and lighting to minimize environmental differences between participants. The dCA experimental setup is illustrated in Figure 2b.

### 2.6 dCA assessment

Participants completed a rhythmic breathing protocol to induce blood pressure changes following a cycle of 6 breaths per minute (5 s in/5 s out). Forced oscillation at 0.1 Hz was chosen as dCA typically functions in the low frequency range (0.07–0.2 Hz) due to the slow nature of vasomotor adaptation and analyzing a fixed frequency reduces variability associated with spontaneous oscillations and improves signal‐to‐noise characteristics. All analyses were completed at this set frequency of 0.1 Hz rather than frequency ranges, as recommended in the recent White paper (Panerai et al., 2023). Participants were asked to demonstrate adherence to this breathing protocol in a short 15–20 s practice period and given a timer that kept track of their inhalation and exhalation. Additionally, participants were instructed to maintain steady shallow breathing and avoid large inhalation and exhalations to minimize changes in PetCO_2_. Participants completed 10 min of quiet, seated rest prior to dCA measurements. Both the input (ABP) and output (HbO) were recorded continuously during the 6 min of rhythmic breathing. Markers for fNIRS and ABP on LabChart were synchronized to time align the two recordings for measurement of dCA using TFA.

Using HbO as an index of flow assumes that metabolic demand remains constant and other physiological factors, such as blood volume and hematocrit, do not significantly contribute to HbO values (Fantini et al., 2016; Fantini & Sassaroli, 2020). During seated, quiet rest, there should be minimal changes in these confounding factors that may allow HbO to be interpreted as CBF, and these two factors have been correlated in animal models (Soul et al., 2000; Tsuji et al., 2000). Using finger photoplethysmography, the sensor in the Finometer finger cuff detects oscillations of the light due to cardiac pulsatility applied to the arterioles measured, allowing for measurements of ABP if the radius of the arterioles is kept constant using the pressure controller unit built into the cuff. ABP measured by this method can be used as a surrogate for cerebral perfusion pressure given that intracranial pressure (ICP) does not change. With seated, rhythmic breathing, no changes in ICP in this population for this study were expected.

Gain, phase‐shift, and coherence were assessed at 0.1 Hz, which has been used in previous studies (Diehl et al., 1998). A high‐pass filter was applied as the cerebrovascular system buffers against slow hemodynamic oscillations, while high frequency responses pass unfiltered to the cerebrovasculature (Wang et al., 2018). ABP and fNIRS data were processed and analyzed using the Cerebral Autoregulation Research Network (CARNet) MATLAB analysis template. fNIRS data were preprocessed with the MATLAB based program HOMER 3 (v.1.80.2, R2017b) (Huppert et al., 2009). Full details for the pre‐processing pipeline for fNIRS and dCA data can be found in File S1.

#### 2.6.1 Neurocognitive battery

Fluid cognitive abilities associated with aging and Mets (Murman, 2015) were assessed with a battery of neurocognitive tasks, including tests of attention, working memory, and executive function, which are known to impaired in MetS (Gosalia et al., 2025) and can be improved with dietary nitrate supplementation (Thompson et al., 2015). Participants completed the digit symbol coding, digit symbol copy, Flanker, and GoNoGo tasks. The digit symbol coding task assessed working memory capacity. The Digit symbol copy assessed processing speed (Wechsler, 2019). The Flanker task assessed executive functioning (interference) (Eriksen, 1995). Lastly, the GoNoGo task assessed executive functioning (inhibition) (Meule, 2017). Information about each task has been previously detailed (Gosalia et al., 2025).

### 2.7 Plasma nitrate and nitrite analysis

Venous blood samples were collected into sodium heparin tubes (6‐mL sodium heparin tubes, BD Vacutainer, Franklin Lakes, NJ, United States) and immediately centrifuged at 3000 rcf (3000 g) and 4°C for 4 min. Plasma was aliquoted and stored in a −80°C freezer for later analysis. The ENO‐20 analyzer was used to measure plasma NO_3_^−^ and NO_2_^−^ concentrations (sensitivity of 0.1 pmol for NO_3_^−^ and NO_2_^−^) according to the manufacturer's protocol.

#### 2.7.1 Statistics

The primary outcome was global frontal dCA. Secondary outcomes included cognitive performance. Exploratory outcomes included region‐specific dCA metrics and associations between plasma nitrate and dCA measures. Given the pilot nature of the study, statistical analyses were considered exploratory, and findings should be interpreted as hypothesis‐generating.

Shapiro–Wilk tests were administered to determine normality of data, and variables that violated test assumptions were analyzed using non‐parametric tests. Outliers outside of cook's distances were removed. Baseline demographics were compared using one‐way analysis of variance (ANOVA) tests between individuals receiving BRJ and those receiving PLB for the 4‐week intervention.

To investigate the effect of 4 weeks of BRJ supplementation on dCA, plasma nitrate and nitrite, and neurocognitive battery performance, a two‐way repeated measures ANOVA was utilized with within‐subjects factor of “Time” with two levels (Baseline, Post‐Intervention) and between‐subjects factor of “Treatment” with two levels (BRJ, PLB). Significant Time × Treatment interaction effects were followed up with Bonferroni‐corrected pairwise comparisons, with partial eta‐squared ($\eta_{p}^{2}$) reported as the effect size. For the primary aim, dCA values were averaged across all the channels to create a global frontal lobe value. The main outcomes for dCA were changes in TFA derived gain and phase. A decrease in gain and an increase in phase would reflect improved dCA. An exploratory aim was to assess the effect of 4 weeks of BRJ supplementation on dCA in each region separately, to determine if effects are region specific, as analysis of the frontal region holistically may obfuscate region‐specific changes. Independent samples *t*‐tests were used to compare delta dCA gain and phase from baseline to post 4‐week intervention between BRJ and PLB groups for each region. Regions included—“left dlPFC”, “right dlPFC”, “left mPFC”, “right mPFC”, “left PMC”, “right PMC”, “left OFC”, “right OFC”. No multiplicity correction was applied because analyses were exploratory.

For bivariate correlation analyses, data were first collapsed between both groups to determine associations between changes in dCA parameters, plasma nitrate, and nitrate, and neurocognitive battery performance. Data was then stratified by treatment to determine associations separately in those that received BRJ from those that consumed PLB. Normality and assumptions were assessed, and Pearson correlations were utilized.

Values in tables are reported as mean ± standard deviation unless otherwise stated. All hypothesis tests were 2‐sided and *α* = 0.05. All analyses were performed on SPSS software version 28 (IBM Corp.). Statistical figures were prepared using GraphPad Prism (v. 10, GraphPad Software, San Diego, CA).

## 3 RESULTS

### 3.1 Palatability and feasibility

Of the 22 participants enrolled in the study, there was a 100% completion rate with no participants withdrawing from the study. Compliance was high, with all participants reporting full adherence based on bottle return and verbal confirmation.

Results from the palatability and acceptability questionnaire are reported in Table **1**. All participants completed the survey. Overall, the palatability of the drink was rated as above average (mean: 6.2 ± 2.6), and 55% of the participants indicated that they enjoyed the drink. In addition, participants were overall neutral to the taste (mean: 5.7 ± 2.8) and aftertaste (mean: 5.6 ± 2.7) of the juice. Approximately 82% of participants described the aftertaste of the juice as neutral or bad. Results from the translational questionnaire are reported in Table 2. With incorporating nitrate into their diet, on a scale of 1–5 in which 1 represented very unlikely, 3 represented neutral, and 5 represented very likely, participants were unlikely to be neutral to the idea of adding BRJ to their diet prior to knowing the health‐related benefits (mean: 2.3 ± 1.3); however, they were more likely to be so when knowing the health‐related benefits (mean: 4.1 ± 1.2). The cost of the juice may be a barrier as participants were overall unlikely to be neutral to purchasing the James White brand for $1.70 a bottle (mean: 2.7 ± 1.4). Lastly, approximately 14% of participants reported unintended side effects, primarily gastrointestinal distress and discomfort; however, no adverse events were reported.

**TABLE 1:** Subjective taste and palatability of beetroot juice.

Item	MetS (*N* = 22)
Perception	
Taste	5.7 ± 2.8
Smell	6.5 ± 2.0
Texture	7.0 ± 2.5
Color	7.5 ± 2.5
Aftertaste	5.6 ± 2.7
Overall experience	6.2 ± 2.6
Taste profiles	
Sweetness	5.6 ± 2.4
Bitterness	2.0 ± 2.4
Sourness	2.1 ± 2.3

*Note*: A score of 0 = highly unpleasant, 5 = neutral, 10 = highly pleasant. Placebo and nitrate‐rich BRJ drink had the same taste profile.

**TABLE 2:** Subjective reports on the translational potential of beetroot juice.

How likely are you to…	MetS (*N* = 22)
Incorporate BRJ into your own diet WITHOUT knowing potential health benefits?	2.3 ± 1.3
Incorporate BRJ into your own diet WITH knowing potential health benefits?	4.1 ± 1.2
Recommend this juice to someone	4.1 ± 1.2
Purchase this juice knowing the price is $1.70 a bottle	2.7 ± 1.4

*Note*: A score of 1 = highly unlikely, 2 = unlikely, 3 = neutral, 4 = likely, 5 = highly likely.

### 3.2 Subject demographics

Of the 22 participants that were randomized, 10 individuals (Female, *n* = 5) received the placebo intervention, and 12 participants (Female, *n* = 10) received the nitrate‐rich intervention. Although not significant (*p* = 0.10), there was an imbalance in the proportion of men and women assigned to each group because sex was not accounted for during randomization. All 22 participants completed the entirety of the study protocol, and all data were of sufficient quality for analysis in the final dataset. The consort diagram is provided in Figure 1.

Participant characteristics are presented in Table 3 (demographics, cardiometabolic variables, and baseline dCA) and Table 4 (cognitive function) and reported as mean ± SD. Baseline differences between groups were observed in HDL‐C (BRJ: 60 ± 9 mg/dL; PLB: 45 ± 4 mg/dL, *p* < 0.001) and triglycerides (BRJ: 133 ± 53 mg/dL; PLB: 198 ± 89 mg/dL, *p* = 0.049). All other variables listed in Tables 3 and 4 were not significantly different between groups (all *p* > 0.15).

**TABLE 3:** Baseline subject demographics, cardiometabolic risk factors, and dCA parameters between individuals receiving BRJ compared to PLB.

	PLB (*n* = 10)	BRJ (*n* = 12)	*p*
Age	64 ± 5	68 ± 8	0.09
Sex	5F/5M	10F/2M	
BMI (kg/m^2^)	31.6 ± 4.6	29.9 ± 3.8	0.34
Waist circumference (cm)	111 ± 11	104 ± 8	0.06
SBP (mmHg)	131 ± 11	133 ± 18	0.74
DBP (mmHg)	71 ± 7	72 ± 10	0.79
Glucose (mg/dL)	104 ± 14	105 ± 21	0.86
Triglycerides (mg/dL)	198 ± 89	133 ± 53	0.049
HDL‐c (mg/dL)	45 ± 4	60 ± 9	<0.001
Education^a^	10.0 ± 1.4	10.6 ± 1.2	0.32
BDI‐II score	3.5 ± 3.5	1.6 ± 2.0	0.15
MMSE score	29.6 ± 0.5	29.3 ± 0.6	0.52
Baseline Plasma Nitrate (uM)	43.4 ± 17.1	39.1 ± 15.6	0.54
Baseline Plasma Nitrite (nM)	0.10 ± 0.02	0.12 ± 0.06	0.40
Baseline dCA Global Frontal Gain (HbO/mmHg)	0.0046 ± 0.0010	0.0058 ± 0.0028	0.23
Baseline dCA Global Frontal Phase (degrees)	34 ± 29	15 ± 18	0.08

^a^ Education was based on a 1–12 scale of highest degree attained, where 5 = high school degree and 9 = college degree (BA or BS).

**TABLE 4:** Baseline neurocognitive performance between individuals receiving BRJ compared to PLB.

	PLB	BRJ	*p*
*n*	10	12	
Flanker accuracy (%)	97.3 ± 4.0	97.4 ± 2.7	0.90
Flanker RT (ms)	539 ± 30	539 ± 52	0.99
Flanker conflict cost	70 ± 24	59 ± 21	0.27
GoNoGo accuracy (%)	94.1 ± 4.4	93.4 ± 4.5	0.73
Digit Symbol Coding Score	36 ± 8	32 ± 6	0.22
Digit Symbol Copy Score	75 ± 17	70 ± 14	0.53

### 3.3 Plasma nitrate and nitrite concentrations

**FIGURE 3:**
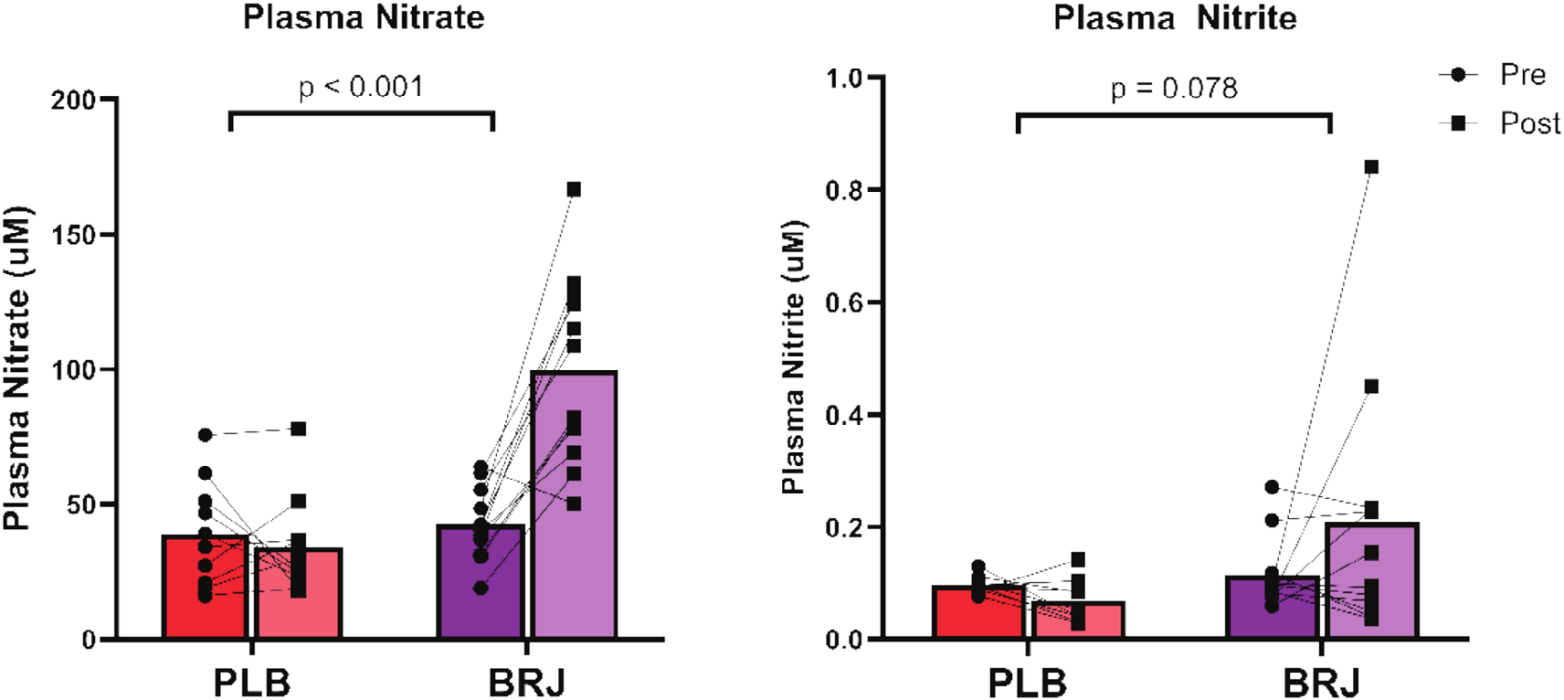
Plasma nitrate and nitrite (uM). Changes in plasma nitrate and nitrite following 4 weeks of nitrate‐rich beetroot juice consumption (BRJ) compared to placebo drink (PLB).

At baseline, there were no significant differences in plasma nitrate (*p* = 0.54) or nitrite (*p* = 0.39) between groups. Following 4 weeks of beetroot juice supplementation, there was a significant Time × Treatment interaction for changes in plasma nitrate concentrations (*p* < 0.001, $\eta_{p}^{2}$ = 0.56), however, there was no Time × Treatment interaction for changes in plasma nitrite (*p* = 0.077, $\eta_{p}^{2}$ = 0.15). Changes in plasma nitrate were significantly greater in individuals that received BRJ compared to PLB following 4 weeks (Δ BRJ: 57 ± 34 uM; Δ PLB: −5 ± 21 uM, *p* < 0.001), however, changes in plasma nitrite concentrations were nonsignificant (Δ BRJ: 93 ± 241 nM; Δ PLB: −29 ± 43 nM, *p* = 0.077), as presented in Figure 3.

#### 3.3.1 BRJ and dCA

**FIGURE 4:**
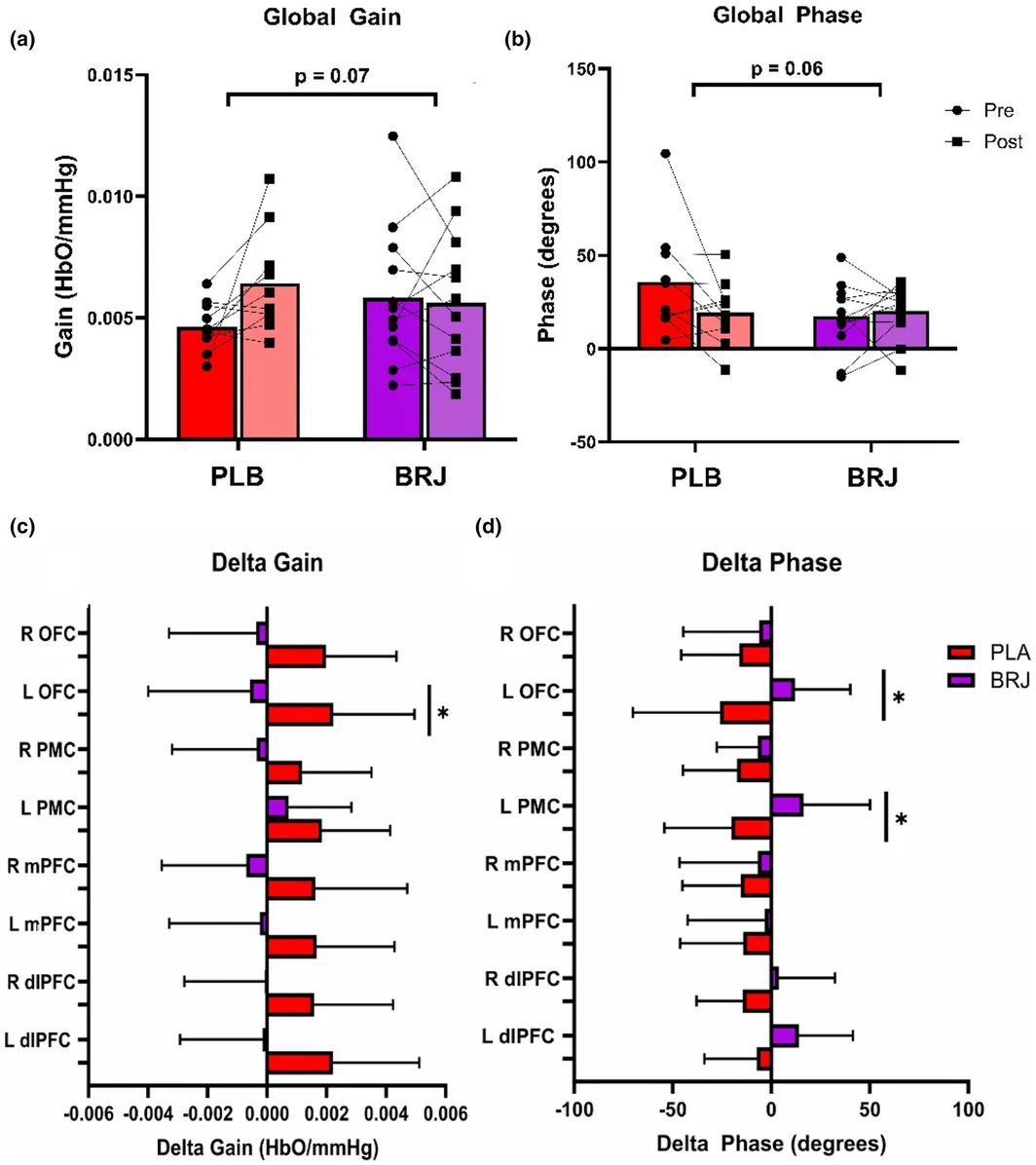
Effect of 4‐weeks of nitrate supplementation on indices of cerebral autoregulation. Changes in gain (a) and phase (b) with BRJ versus PLB from baseline with all regions averaged. Delta changes for gain (c) and phase (d) are also shown between treatment and baseline for each region. *p < 0.05.

There were no significant differences in coherence between baseline, and the 4‐week follow up visit (Baseline: 0.84 ± 0.16, Post: 0.81 ± 0.18, *p* = 0.25), nor between the BRJ and PLB groups at either timepoint (Baseline: BRJ: 0.86 ± 0.16, PLB: 0.84 ± 0.15, *p* = 0.74; Post‐Intervention: BRJ: 0.78 ± 0.22, PLB: 0.82 ± 0.16, *p* = 0.62). Primary analyses showed no significant effect of “Time” (*p* = 0.15, $\eta_{p}^{2}$ = 0.10) or “treatment” (*p* = 0.83, $\eta_{p}^{2}$ = 0.002) or “region × treatment” (*p* = 0.07, $\eta_{p}^{2}$ = 0.15) on global frontal dCA gain (Figure 4a). Exploratory analyses were completed to compare “treatment” effects on dCA gain separately for each region using the delta between the 4‐week follow up visit and the baseline visit. Across all eight regions interrogated, there was a significant “treatment” effect for the L OFC (Δ BRJ: −0.0006 ± 0.0034; Δ PLB: +0.0023 ± 0.0021; *p* = 0.05, *d* = 1.02) and moderate‐to‐large effect sizes were observed in the L dlPFC (*p* = 0.066, *d* = 0.83), R mPFC (*p* = 0.083, *d* = 0.77), R OFC (*p* = 0.055, *d* = 0.89) regions. Exploratory analyses of region specific changes in dCA gain between BRJ and PLB are presented in Figure 4c. Exploratory analyses were not adjusted for multiple regional comparisons and should be interpreted as hypothesis‐generating findings.

Primary analyses showed no significant effect for “Time” (*p* = 0.18, $\eta_{p}^{2}$ = 0.09), “treatment” (*p* = 0.23, $\eta_{p}^{2}$ = 0.07) or “region × treatment” (*p* = 0.06, $\eta_{p}^{2}$ = 0.16) on global frontal dCA phase (Figure 4b). Exploratory analyses were also completed to compare “treatment” effects on dCA phase separately for each region using the delta between the 4‐week follow up visit and the baseline visit. Across all eight regions interrogated, there was a significant “treatment” effect for the L PMC (Δ BRJ: +16.3 ± 21.8°; Δ PLB: −20.0 ± 25.8°; *p* = 0.020, *d* = 1.52) and the L OFC (Δ BRJ: +12.1 ± 32.2°; Δ PLB: −26.0 ± 34.5°; *p* = 0.023, *d* = 1.14) and moderate‐to‐large effect sizes were observed in the L dlPFC (*p* = 0.08; *d* = 0.79). Exploratory analyses of region specific changes in dCA phase between BRJ and PLB are presented in Figure 4d and represent hypothesis‐generating findings.

### 3.4 BRJ and neurocognitive battery performance

No significant effects of Time, Treatment, or Time × Treatment were observed for any cognitive outcome (all *p* > 0.11; Table 5).

**TABLE 5:** Effects of 4 weeks of BRJ supplementation on neurocognitive performance.

	PLB (*n* = 10)		BRJ (*n* = 12)		*p*
	Pre	Post	Pre	Post	Time × treatment
Flanker accuracy (%)	97.3 ± 4.0	97.8 ± 3.4	97.4 ± 2.7	95.0 ± 7.0	0.10
Flanker RT (ms)	539 ± 30	524 ± 19	539 ± 52	526 ± 86	0.90
Flanker conflict cost	70 ± 24	68 ± 20	59 ± 21	50 ± 29	0.47
GoNoGo accuracy (%)	94.1 ± 4.4	96.5 ± 3.3	93.4 ± 4.5	94.3 ± 5.2	0.67
Digit Symbol Coding Score	36 ± 8	36 ± 5	32 ± 6	35 ± 5	0.14
Digit Symbol Copy Score	75 ± 17	74 ± 14	70 ± 14	73 ± 16	0.56

*Note*: Repeated measures ANOVA revealed no time × treatment interactions for any assessment.

### 3.5 Exploratory correlation analyses

**FIGURE 5:**
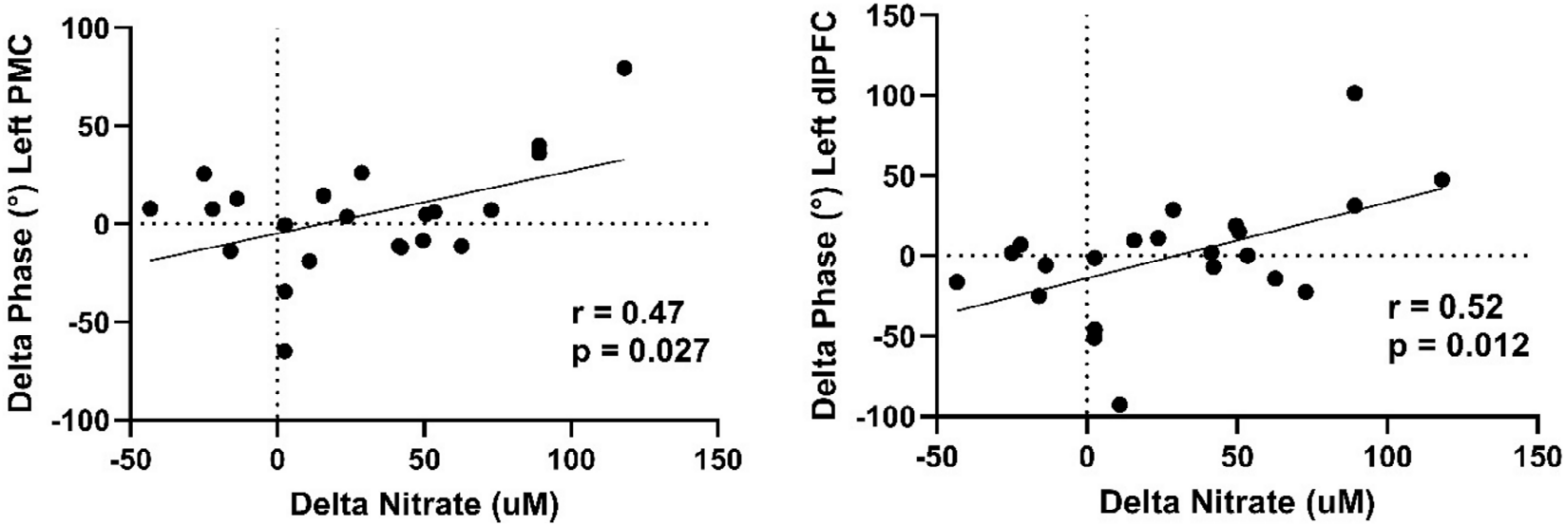
Association between changes in plasma nitrate and changes in TFA‐derived phase in the left premotor cortex (L PMC) and left dorsolateral prefrontal cortex (L dlPFC) collapsed across all participants.

Exploratory bivariate correlation analyses revealed significant associations between changes in plasma nitrate and changes in phase of the left dlPFC (*r* = 0.47, *p* = 0.027) and left PMC (*r* = 0.52, *p* = 0.012) when collapsing the two groups (Figure 5) and should be interpreted cautiously. When assessing these associations in only the BRJ group, a significant association between changes in plasma nitrate and changes in phase of the left dlPFC remained (*r* = 0.60, *p* = 0.039); however, the association with plasma nitrate and phase of the left PMC was attenuated and no longer significant (*r* = 0.493; *p* = 0.10). No other significant associations were observed between changes in plasma nitrate/nitrite, neurocognitive battery performance, or dCA when collapsing both groups for analysis, or when stratifying by treatment group.

## 4 DISCUSSION

In this pilot double‐blind, placebo‐controlled randomized trial, we evaluated the feasibility of 4 weeks of once‐daily nitrate‐rich beetroot juice supplementation and explored its associations with dynamic cerebral autoregulation and cognition in older adults with metabolic syndrome. The intervention was feasible, well tolerated, and increased circulating plasma nitrate concentrations, confirming compliance. Primary analyses revealed no significant effects on global frontal dCA or cognitive performance. However, exploratory region‐specific analyses identified differences in dCA metrics within select left frontal regions, and exploratory correlations suggested modest associations between changes in plasma nitrate and regional dCA phase. Given the modest sample size and exploratory nature of these analyses, these findings should be interpreted cautiously and as hypothesis‐generating rather than evidence of efficacy.

### 4.1 Feasibility of chronic nitrate‐rich beetroot juice supplementation

Four weeks of inorganic nitrate‐rich beetroot juice supplementation was safe and feasible in older adults with MetS. Adherence to the study was high and participants self‐reported that the palatability of the juice was largely neutral. Adherence to the juice may have been strong because participants rated their likelihood to consume the drink higher if there were known health benefits associated with chronic consumption. Although the overall taste of the juice was rated as neutral, it is encouraging that participants were willing to consume the juice if there was potential for health benefits. Price may be a barrier for population level implementation, and strategies to make beetroot juice affordable may improve the appeal of daily consumption of nitrate‐rich beetroot juice. No adverse events from the intervention were reported; however, some unintended side effects were observed that included gastrointestinal distress and discomfort. Further investigation into the causes of GI discomfort in some of the participants, and strategies to minimize these side effects are needed. Overall, 4 weeks of beetroot juice supplementation was feasible and palatable in this cohort of older adults and may suggest that long‐term clinical trials implementing beetroot juice supplementation can be achievable.

### 4.2 dCA, cognition, and potential mechanisms

Given participant's high enthusiasm to consume beetroot juice if health benefits were associated with daily consumption, determining benefits towards cognitive and cerebrovascular health was the next question to address. Although our primary analysis revealed no significant effects of BRJ on dCA gain or phase when the frontal region was analyzed holistically, exploratory analyses revealed that improvements in dCA may be region specific in the frontal lobe. Although changes in frontal dCA holistically were insignificant, both dCA gain and phase trended towards improvement with nitrate‐rich BRJ (*p* = 0.06 and 0.07). A small sample size may have contributed to low power to observe a significant finding. Alternatively, improvements in dCA may have been more regional, and a holistic measure of frontal dCA could have obfuscated regional improvements given regions that did not improve would be averaged. Previous investigations do suggest that regional dCA impairments occur in brain regions more susceptible to atrophy, such as the prefrontal cortex which has a high density of white matter (Aoi et al., 2012). Thus, these atrophy‐prone regions may have a greater ceiling for improvement.

With our exploratory aims, we observed increased phase and decreased gain in the left premotor and orbitofrontal cortex, which reflect better synchrony between rises in blood pressure and fNIRS‐derived HbO, as well as dampening of blood pressure oscillations. These frontal regions (premotor and orbitofrontal cortex) are highly perfused and susceptible to aging and cardiometabolic risk factors, according to the frontal aging hypothesis (Raz et al., 2005; West, 1996). Four weeks of dietary nitrate supplementation, although speculative, may have partially ameliorated cerebral microvascular endothelial dysfunction to improve dCA. However, these exploratory findings may be partially explained by baseline differences in HDL‐c and triglycerides between the BRJ and PLB groups as post‐hoc sensitivity analyses including these variables as covariates slightly attenuated significant findings (*p* = 0.06–0.08; data not shown). Because our regional analyses were exploratory and hypothesis‐generating, we discuss the results in the context that these significant findings need to be explored more thoroughly in an adequately powered RCT rather than suggesting physiological efficacy of nitrate supplementation. However, we recognize the imbalance in these variables may introduce confounding effects in our analyses.

A few other investigations outside of the current pilot randomized controlled trial have examined dietary nitrate supplementation and dCA. A study by Fan et al. (2019) found that 7 days of dietary nitrate supplementation (0.1 mmol·kg^−1^·day^−1^) increased plasma nitrate concentrations and improved cerebrovascular reactivity and dCA gain as measured by TFA with transcranial doppler ultrasound and beat‐to‐beat blood pressure in healthy adults. Our findings may suggest potential effects of dietary nitrate supplementation on dCA for older adults with MetS. However, a study by Horiuchi et al. (2022) found no improvements in dCA with 4 days of inorganic dietary nitrate in young healthy men. Discrepancies between studies may be attributable to a younger, healthier population and shorter intervention duration in the study by Horiuchi et al. compared to the current study, which investigated an older and less healthy population with baseline endothelial dysfunction (Gosalia et al., 2025) that may be more responsive to intervention. Findings from the current study add to the scarcity of investigations on this topic and provide hypothesis‐generating data to further explore whether dietary nitrate improves dCA in specific frontal regions in MetS.

Interestingly, exploratory analyses suggested that differences in dCA metrics following nitrate‐rich BRJ supplementation were more consistently observed in left frontal regions, including the left orbitofrontal cortex, premotor cortex, and dorsolateral prefrontal cortex. The apparent lateralization of these findings may reflect underlying hemispheric differences in vascular vulnerability or functional specialization (Mohammadi et al., 2025), although this observation should be interpreted cautiously given the modest sample size and multiple regional comparisons. Future adequately powered studies using multimodal neuroimaging approaches are needed to determine whether dietary nitrate supplementation exerts regionally specific cerebrovascular effects and whether such effects preferentially occur in left frontal regions.

In the current study, no changes in cognitive performance were detected. Although previous studies have also observed no improvements in executive functioning or memory after short‐term nitrate supplementation (Jackson et al., 2020; Kelly et al., 2013), Aliahmadi et al. (2021) demonstrated improvements in sustained attention and processing speed after 8 weeks of raw red beetroot consumption (100 g) in middle‐aged T2D, which may indicate that a longer intervention period is necessary to observe changes in cognition. Previous investigations that have observed improvements in cognition with dietary nitrate included more diseased populations with baseline deficits in cognitive functioning and a longer duration of supplementation (Bråthen et al., 2022; Jung et al., 2025). According to the vascular contributions to cognitive impairment (VCID) framework, vascular dysfunction and brain structure abnormalities precede cognitive decline (Zlokovic et al., 2020). In line with VCID, it is then likely that improvements in cognition require longer‐term vascular remodeling and structural brain changes, suggesting changes in physiology (dCA) may precede cognitive benefits. Thus, longer‐term trials will be necessary to assess if improvements in dCA precede improvements in cognitive performance following dietary nitrate supplementation in older adults with MetS. Lastly, the current pilot trial had a small sample size and interindividual variability may have masked subtle cognitive impairments, necessitating a greater sample size in future studies.

### 4.3 Plasma nitrate and nitrite and dCA

We observed increases in plasma nitrate following 4 weeks of BRJ, confirming adherence to the intervention. However, plasma nitrite was not significantly increased, possibly reflecting high interindividual variability and its short half‐life (Wang et al., 2013). In the current study, there was a 24‐h period separating participants' last juice consumption and post‐intervention assessments to test the chronic effects of juice consumption. There is a scarcity of literature on resting plasma nitrate and nitrite concentrations following chronic beetroot juice supplementation. However, Miller et al. observed increases in plasma nitrate and nitrite from baseline and acute supplementation values following 4‐weeks of beetroot juice consumption (380 mg NO_3−_/70 mL) (Miller et al., 2024), suggesting chronic beetroot juice supplementation may increase plasma nitrate and nitrite concentrations. This increase may be lost with extended duration following the final dose, as observed in this current study. Additionally, literature suggests adults may be responders versus nonresponders to increases in plasma nitrite via reduction of nitrate to nitrite in the oral cavity due to differences in microbial composition and diversity (Goh et al., 2022). MetS exhibits a disrupted oral microbiota (Prince et al., 2023), and thus some participants may have had a lower capacity to reduce nitrate to nitrite; however, this warrants further investigation.

Although only plasma nitrate was increased following 4 weeks of supplementation in this study, we observed a significant positive association between changes in plasma nitrate and changes in dCA phase of the left PMC and dlPFC. This association was stronger for the L dlPFC in just the group that received the BRJ, in which plasma nitrate levels were perturbed, compared to all participants collectively (*r* = 0.60 vs. *r* = 0.47). This exploratory finding may suggest that increasing NO bioavailability, reflected by increased plasma nitrate, is associated with improved dCA in older adults with MetS. It is debatable whether increases in plasma nitrate reflect increased NO bioavailability; there is evidence that plasma nitrite, but not nitrate, reflects NO production from endothelial nitric oxide synthase (eNOS) in humans (Kim‐shapiro & Gladwin, 2015), as well as endothelial dysfunction in humans (Kleinbongard et al., 2006). It is plausible that nitrate can reflect eNOS activity as mice that overexpress eNOS are shown to have elevated levels of both plasma nitrate and nitrite (Duranski et al., 2006). A future adequately powered trial is needed to confirm if regional improvements in dCA are attributable to increased plasma nitrate and NO bioavailability.

MetS is a pathophysiological state that coincides with pathological conditions affected by reduced NO bioavailability (Litvinova et al., 2015) and may be a population that benefits from exogenous sources such as dietary nitrate. There is a dearth of studies that have investigated dietary nitrate supplementation in MetS on either systemic or cerebral vascular function in humans. However, dietary nitrate supplementation has been effective in increasing plasma nitrate and nitrite and improving systemic macrovascular flow‐mediated dilation and arterial stiffness in populations akin to MetS, including hypercholesteremia (Velmurugan et al., 2016), overweight and obesity (Joris & Mensink, 2013), and hypertension (Kapil et al., 2015). The current study extends the literature with a potential benefit of dietary nitrate to MetS for dCA; however, the efficacy of nitrate supplementation on dCA needs to be further tested in an adequately powered study to confirm the findings of this pilot trial.

### 4.4 Assessment of dCA using fNIRS

A strength of the current study is the use of functional near‐infrared spectroscopy (fNIRS) combined with transfer function analysis to assess regional dCA. Unlike transcranial Doppler ultrasound, which primarily measures velocity in large conduit arteries, fNIRS provides regional information regarding cortical oxygenation and may better reflect microvascular autoregulatory responses. This approach enabled identification of potential region‐specific differences in dCA that may not be detectable using global measures alone. Although the current literature utilizing fNIRS for dCA measurements with the transfer function analysis approach is limited, previous work supports the utility of fNIRS‐derived signals in assessing cerebrovascular regulation (Eleveld et al., 2021; Sainbhi et al., 2023; Zweifel et al., 2010). It is important to recognize that fNIRS‐derived [HbO] is an indirect surrogate of microvascular cerebral blood flow and may be influenced by extracranial contamination, limited tissue penetration, cerebral blood volume changes, and variability in optode placement (Huo et al., 2021). Although post‐processing of data was carefully conducted to remove noise and extracerebral components, we recognize that a lack of measurement of extracranial blood flow is a limitation in our approach. The fNIRS‐derived HbO signal reflects relative changes in microvascular oxyhemoglobin. A future study that compares responses between fNIRS‐derived HbO and direct measures of cerebral blood flow would help strengthen the utility of the fNIRS‐derived HbO signal as an index for microvascular cerebral blood flow. Future studies incorporating complementary neuroimaging modalities that include direct measurements of cerebral blood flow, as well as measurements of extracerebral blood flow, are needed to confirm these findings.

### 4.5 Experimental considerations and limitations

The primary limitation of this study is the modest sample size, which limits statistical power and increases the likelihood of both type I and type II error. Accordingly, all findings should be interpreted as exploratory and hypothesis‐generating. Next, multiple exploratory regional and correlational analyses were performed without adjustment for multiplicity, increasing the risk of false‐positive findings. Additionally, significant baseline differences in HDL cholesterol and triglycerides were present between groups despite randomization. Because these factors are associated with vascular function and nitric oxide bioavailability, it is possible that they influenced individual responsiveness to nitrate supplementation. However, given the pilot nature of this study and limited sample size, we were unable to determine whether baseline lipid status modified treatment effects. Although insignificant, there was a greater proportion of women in the BRJ group. It is possible women may be differently responsive to BRJ supplementation and may be a potential source of variability (Wickham & Spriet, 2019); however, the current study was not powered to thoroughly investigate sex differences and warrants further investigation. Next, we only observed significant increases in plasma nitrate and not nitrite at 24 h after the final dose consumption. Future investigations should measure plasma nitrate and nitrite periodically throughout the 4‐week intervention and take both acute and extended measurements after the final dose to account for the short half‐life or incomplete clearance of plasma nitrate and nitrite in the blood. For dCA assessment, we did not measure PetCO_2_ and cannot rule out the possibility of reductions in PetCO_2_ on decreasing fNIRS‐derived cerebral blood flow velocity and biasing TFA metrics, including artificially increased phase and decreased gain. To minimize this possibility, participants were instructed and monitored to maintain steady shallow breathing of 6 breaths/min throughout testing, and identical testing procedures were used at both study visits. Furthermore, because participants served as their own controls over time, the primary analyses focused on within‐subject longitudinal changes rather than cross‐sectional group differences. This design reduces, although does not eliminate, the likelihood that between‐visit differences in respiratory behavior fully account for the observed findings. However, the absence of arterial CO_2_ measurements limits physiological interpretation, and future studies should incorporate continuous PetCO_2_ monitoring to better account for potential influences of ventilation on cerebral hemodynamics. Lastly, fNIRS provides an indirect measure of cerebral hemodynamics and is subject to known technical limitations.

#### 4.5.1 Future directions

Future studies should build on these preliminary findings by employing adequately powered randomized controlled designs with stratified randomization and longer intervention durations to determine whether dietary nitrate supplementation influences frontal or region‐specific dCA in older adults with MetS. Sample size estimates for a future adequately powered clinical trial (*β* = 0.80) with appropriate multiple comparison correction (*α* = 0.00625) that is based on observed effect sizes from regional dCA analyses in the current study indicate 40 individuals with equal allocation between groups would be sufficient. Incorporating multimodal neuroimaging techniques and mechanistic biomarkers of endothelial function and nitric oxide bioavailability may help clarify the physiological pathways underlying any observed effects. Additionally, longer‐term studies are needed to determine whether potential cerebrovascular changes translate to meaningful improvements in cognitive function or reduced risk of cognitive decline.

## 5 CONCLUSIONS

In conclusion, 4 weeks of nitrate‐rich beetroot juice supplementation was feasible, well tolerated, and increased circulating plasma nitrate concentrations in older adults with metabolic syndrome. No significant effects were observed in primary analyses of dynamic cerebral autoregulation of the frontal region holistically or with cognitive performance. However, exploratory analyses suggested potential region‐specific differences in autoregulatory responses within select frontal regions. These findings should be interpreted as hypothesis‐generating and may inform the design of future adequately powered trials investigating the effects of dietary nitrate supplementation on dCA and cognitive health in individuals with cardiometabolic risk.

## AUTHOR CONTRIBUTIONS

**Jigar Gosalia:** Conceptualization; data curation; formal analysis; funding acquisition; investigation; methodology; validation; visualization. **Jocelyn M. Spicuzza:** Conceptualization; data curation; formal analysis; investigation; project administration; resources; supervision. **Christine Bowlus:** Conceptualization; investigation; supervision. **Annabelle Brinkerhoff:** Data curation; formal analysis; investigation. **Stephen R. Baker:** Data curation; formal analysis. **Juan Jan Qiu:** Investigation; resources; supervision. **Daniel B. Kim‐Shapiro:** Data curation; formal analysis; resources; supervision. **Jim A. Pawelczyk:** Conceptualization; data curation; formal analysis; supervision. **David N. Proctor:** Conceptualization; investigation; project administration; resources; supervision.

## FUNDING INFORMATION

The author(s) declare financial support was received for the research, authorship, and/or publication of this article. This research was supported by the National Center for Advancing Translational Sciences, National Institutes of Health (NIH) through grant UL1 TR002014. The content is solely the responsibility of the authors and does not necessarily represent the official views of the NIH. The project described was also supported by the Huck Endowment for Nutritional Research in Family and Community Medicine at Penn State College of Medicine and University Park.

## CONFLICT OF INTEREST STATEMENT

The authors have no financial or non‐financial conflicts of interest to disclose.

## Supporting information


File S1.

## Data Availability

Source data can be accessed at: https://github.com/JigarGosalia/BRJ‐and‐dynamic‐cerebral‐autoregulation‐data‐repository.git.
