# Supplementary material for: A pilot randomized controlled trial of the effects of dietary nitrate supplementation on dynamic cerebral autoregulation in older adults with metabolic syndrome
Source: Physiol Rep. 2026 Aug 25;14(16):e71073. doi: 10.14814/phy2.71073 (PMC13507522; doi:10.14814/phy2.71073)
Supplement: Supplementary file 1 [file PHY2-14-e71073-s001.docx]

**Supplemental File 1 – Methods**

*Functional near infrared spectroscopy (fNIRS) Setup*

NIRScaps were fitted using the Nasium (Nz) and Inium (Iz), along with the left and right pre-auricular points (LPA and RPA) as reference points (located by visual inspection) according to the international 10-20 system to ensure the cap was aligned evenly and similarly between participants. Additionally, the reference point Cz was checked to determine if it aligned equidistantly between Nz and Iz and between LPA and RPA and if the Fpz-Iz line was aligned evenly along the midsagittal plane.

fNIRS recordings were calibrated and captured using the software NIRSite (NIRx, Glen Head NY). The differential pathlength factor (DPF) of the photon path was adjusted to account for differences in skull thickness and cortical depth that result from chronological aging [28]. Signal quality was determined using software NIRSite in which thresholds for coefficient of variation (C.V) to categorize channels were designated as excellent (C.V. < 2.5%), acceptable (C.V. > 2.5% and < 7.5%), critical (>7.5%), and too low (signal absent). Any channels designated as ‘critical’ or ‘too low’ after multiple calibrations were noted and discarded from averaging for each ROI. In our dataset, an average of ~0.8 channels were excluded per participant of the possible 40 channels for both baseline and end of study assessment of dCA. A total of 37 channels were not included for analysis across 1760 possible channels (40 channels x 22 participants x 2 time points), which is equal to ~2.1% total channels rejected. These discarded channels were not included for averaging in either the global frontal lobe value or for the corresponding ROI. All fNIRS signals were recorded with sampling frequency of 4.464 Hz.

*dCA post-processing*

Initiating the preprocessing pipeline, raw signals were converted to optical density units. Channels were then inspected for motion artifacts, and any flagged motion artifact was corrected using a spline interpolation. Corrected data were then bandpass filtered with a low pass filter of 0.5 Hz and a high pass filter of 0.005 Hz to remove noise and slow drifts, respectively. Lastly, pre-processed optical densities were converted to changes in chromophore concentrations using the modified beer-lambert law [39], providing relative changes in HbO and HbR (μM cm). The power spectra of each channel were then visually inspected to confirm a peak at 0.1 Hz.

Following fNIRS data processing, ABP and HbO data were exported at 4.464 Hz to organize in an excel template for dCA analysis using the MATLAB based CARNet transfer function analysis script [26,39]. Because breathing was set at a forced oscillation of 0.1 Hz, data at that set frequency was recorded and used for analyses rather than frequency bands [26]. Default parameters for dCA analysis were taken from the 2023 updated consensus paper by Panerai and colleagues [26]. Data was first spectrally smoothed using a triangular filter, and then each 6-min recording was subdivided into 7 successive windows of 100s length and 50% overlap. Each window was passed through a Hanning window prior to Fourier transformation and the cross spectrum between HbO and ABP was calculated for TFA [26]. Outcome variables included TFA derived gain, phase-shift, and coherence values at 0.1 Hz globally with all channels averaged and for each region of interest. All data in which coherence between ABP and HbO was < 0.35 was excluded from the study. This critical threshold for coherence was chosen since 7 total windows were adopted for data segmentation with Welch’s method at a 99% significance level for TFA analysis [26]**.** Of the 352 total dCA analyses completed (22 participants x 8 regions x 2 timepoints), coherence was < 0.35 for 8 of them (2% of all analyses) and excluded from the final dataset. Phase shift was corrected for cerebral microcirculation effects on waveform morphology that results in a constant negative phase shift by subtracting the mean phase shift from the high frequency range in which dCA is inactive from the individual phase shift values at 0.1 Hz [40].
